# Diagnostic Radiology Exercises

**Published:** 1987-11

**Authors:** Gordon Thomson


### Book Review

### DIAGNOSTIC RADIOLOGY EXERCISES

**Paul R. Goddard**

**198 pages, 136 images and several diagrams, 1987**
**Wright, £9.95 ISBN 07236 0516 5**

This stimulating book is presented as a series of revision exercises for doctors taking the examinations M.R.C.P., F.R.C.S., M.R.C.O.G., and D.M.R.D. It could also usefully be read by many practising radiologists, wishing to pit their skills against the illustrations posed. Eighty cases are presented in groups of five in each group, with answers provided, together with discussion, on the ensuing pages. Specialities covered include General Medicine, Paediatrics, Orthopaedics and General Surgery, and the illustrations are mostly of radiographs of the chest, abdomen and skeleton, with a few ultrasound and isotope scans included.

The cases cover a varied and interesting selection of diseases, and the discussion about each case is packed full of information regarding the differential diagnosis and further management of the patient. These answers are a valuable part of the book, condensing as they often do a summation of helpful detail that one could spend hours searching for in larger text books. A criticism could arise in Case 47, where it would appear that a cerebral angiogram was the first radiological investigation carried out in a patient with a subarachnoid haemorrhage. A CT Scan carried out within, say five days of the onset of symptoms, and the sooner the better, is the usual initial investigation as indeed the valuable figure 47.2 illustrates. A lumbar puncture in this case would be superfluous. A misprint follows in the text, and should read "blood within the ventricular system". One or two other spelling mistakes were noted throughout the book. These are, however, minor points in what must rank as a really splendid book at a very reasonable price, and an invaluable stimulus to those about to take higher examinations, as well as quite a severe test for numerous radiologists already holding the required qualifications. Easy to read, informative and provocative it deserves to have a wide following.

Gordon Thomson

